# 4SCAR2.0: a multi-CAR-T therapy regimen for the treatment of relapsed/refractory B cell lymphomas

**DOI:** 10.1038/s41408-021-00455-x

**Published:** 2021-03-17

**Authors:** Cheng Jiao, Eugene Zvonkov, Xun Lai, Rui Zhang, Yuchen Liu, Yue Qin, Valery Savchenko, Nelly Gabeeva, Tsai-Hua Chung, Liyuan Sheng, Lung-Ji Chang

**Affiliations:** 1 Shenzhen Geno-Immune Medical Institute, 518000 Shenzhen, China; 2 PKU-HKUST Shenzhen-Hong Kong Institution, 518057 Shenzhen, China; 3 National Research Center for Hematology, Moscow, 125167 Russia; 4 The Third Affiliated Hospital of Kunming Medical University, Cancer Hospital of Yunnan, 650118 Kunming, Yunnan China; 5 University of Electronic Science and Technology of China, 611731 Chengdu, Sichuan China; 6 Peking University, Shenzhen Institute, Shenzhen Key Lab Human Tissue Regenerate & Repair, 518057 Shenzhen, China

**Keywords:** Immunotherapy, B-cell lymphoma

Dear Editor,

Chimeric antigen receptor (CAR) gene therapy is a breakthrough technology in treating refractory hematologic malignancies^1–3^. CD19 CAR-T therapy has emerged as a promising treatment for the management of B cell malignancies. However, malignant cell populations of leukemia or lymphoma are highly heterogeneous and tend to diverse into CD19-negative cells after targeted treatment. Antigen escape leading to disease recurrence is the major reason for the failure of currently approved CAR-T cell therapies^4^. Similar to combining different chemotherapy agents in order to overcome chemotherapy resistance, investigators have started combining CAR-T cells targeting different antigens, in order to overcome tumor antigen escape^5,6^. Besides, the associated severe adverse effects, the exhaustion of CAR-T cells in vivo, and the high cost of individualized CAR-T cell preparation are major limitations to the current CAR technology^7^.

Treatment options for relapsed/refractory B cell lymphomas (rrBCLs) such as primary mediastinal B cell lymphoma (PMBCL) and central nervous system (CNS)-involved BCL are limited, and prognosis is generally poor, with an overall response rate (ORR) of 0–25% and 2-year overall survival around 15%^8–10^. Brain metastases are often the final lethal consequence of many aggressive cancers. To improve safety, overcome tumor escape, and prolong in vivo CAR-T efficacy, we have developed a novel multi-CAR-T therapy regimen (4SCAR2.0, fourth-generation safety-designed CAR) to target highly refractory BCLs, which involves a primary, a booster, and an optional consolidation CAR-T infusions targeting multiple antigens based on individual tumor antigen profile. Here we describe our experience with multi-CAR-T cell therapy using an improved fourth-generation CAR design with an excellent safety record in treating patients (pts) with rrBCL.

The current study was approved by the Institutional Review Board of Geno-Immune Medical Institute (GIMI) of Shenzhen, China (GIMI-IRB-17005) and registered at ClinicalTrials.gov (NCT03125577). Pts provided informed consent according to institutional guidelines and the Declaration of Helsinki. Lymphoma pts who have exhausted all available treatments with progressive or stable disease and life expectancy >2 months are enrolled in the study. This 4SCAR2.0 regimen is developed to include a primary, a booster, and/or a consolidation CAR-T infusions targeting multiple tumor antigens. The choice of CAR-T targets is based on immunostaining of CD19, CD22, CD30, GD2, and PSMA of tumor biopsies. Autologous T cells were isolated from apheresis blood mononuclear cells and transduced with an apoptosis-inducible, safety-engineered lentiviral CAR with the following intracellular signaling domains: CD28/CD27/CD3ζ-iCasp9 (4SCAR).

Pts were treated between September 2018 and December 2019. This report includes four pts with rrBCL, three of whom have relapsed after previous chemotherapy and autologous stem cell transplantation. The first pt was diagnosed with PMBCL with a relapsed brain metastasis. The second pt had a relapsed diffuse large B cell lymphoma, and the other two pts had relapsed follicular lymphomas. The details of each case are presented below and summarized in Table 1.

**Table 1:** Patient and treatment characteristics.

Patient #	1	2	3	4
Age at diagnosis, years	32	45	48	61
Underlying lymphoma	PMBCL	DLBCL	FL/DLBCL	FL
Staging at diagnosis/sites of metastatic disease	Stage IV/gastric mucosa, left lung CNS negative	Stage IV/GCB axillary, intrathoracic, retroperitoneal lymph nodes; liver, CNS positive	Stage III/GCB axillary, retroperitoneal, mesenteric lymph nodes, mesentery of the small intestine	Stage IVB/peripheral, intrathoracic, intra-abdominal, retroperitoneal lymph nodes, bone marrow
Previous treatment	DA-EPOCH	R-mNHL-BFM-90, R-DHAP, R-CEAM, ASCT	Obinutuzuab, Benda mustine, R-CHOP, R-DHAP, CEAM, ASCT	R-CHOP, R-DHAP, mNHL-BFM-90, lenalidomide, CEAM, ASCT
Disease status before lymphodepletion	PD	CR	PR	PR
Interventions	#1 infusion on D0: 4SCAR19+30 (2.2 + 2.65 × 10^6^/kg) #2 infusion on D42: 4SCAR19+22 (1.76 + 0.66 × 10^6^/kg) #3 infusion on D69: 4SCAR19-153z (0.75 × 10^6^/kg) #4 infusion on D265: 4SCAR PSMA (0.8 × 10^6^/kg)	#1 infusion on D0: 4SCAR19+22 (0.09 + 0.08 × 10^6^/kg) #2 infusion on D3: 4SCAR19 (0.87 × 10^6^/kg)	#1 infusion on D0: 4SCAR19+22 (4.4 + 2.8 × 10^6^/kg) #2 infusion on D7: 4SCAR-GD2 (0.38 × 10^6^/kg) #3 infusion on D14: 4SCAR19-153z (0.31 × 10^6^/kg)	#1 infusion on D0: 4SCAR19+22 (0.12 + 0.14 × 10^6^/kg) #2 infusion on D13: 4SCAR19-153z + CD22-153z (0.06 + 0.63 × 10^6^/kg)
Maximum CRS/NT (ASTCT 2019)	Grade 1 CRS No NT	Grade 1 CRS No NT	No CRS No NT	No CRS No NT
Initial post-CAR-T response	Day 26 PR	Day 28 CR	Day 22 CR	Day 49 CR
Subsequent follow-up	Day 186 CR Day 725 CCR	Day 323 CCR	Day 453 CCR	Day 290 CCR

*ASTCT* American Society for Transplantation and Cellular Therapy, *PD* progressive disease, *CR* complete response, *CCR* continuous complete remission, *PR* partial response, *DA-EPOCH-R* dose adjusted etoposide, prednisone, vincristine, cyclophosphamide, doxorubicin, and rituximab, *F* female, *GCB* germinal center B cell, *M* male, *PMBCL* primary mediastinal B cell lymphoma, *DLBCL* diffuse large B cell lymphoma, *FL* follicular lymphoma, *R* rituxan, *R-CHOP* rituxan/cyclophosphamide/doxorubicin/vincristine/prednisone, *R-DHAP* rituximab/dexamethasone/cytarabine/cisplatin, *R-CEAM* rituximab/carmustine, etoposide, cytarabine, and melphalan, *G-PEPC* prednisone/etoposide/procarbazine/cyclophosphamide, *ASCT* autologous stem cell transplantation, *CRS* cytokine release syndrome, *NT* neurotoxicity.

All pts received more than one CAR-T infusions targeting CD19, CD22, CD30, GD2, and/or PSMA. Before the first CAR-T infusion, pts received cyclophosphamide/fludarabine chemotherapy conditioning. The infusion dose ranged from 0.06 to 4.4 × 10^6^ CAR-T cells/kg per infusion. The quality of apheresis cells, efficiencies of gene transfer, T cell proliferation, CAR-T dose, and blood CAR copies were quantitatively documented.

**Fig. 1:**
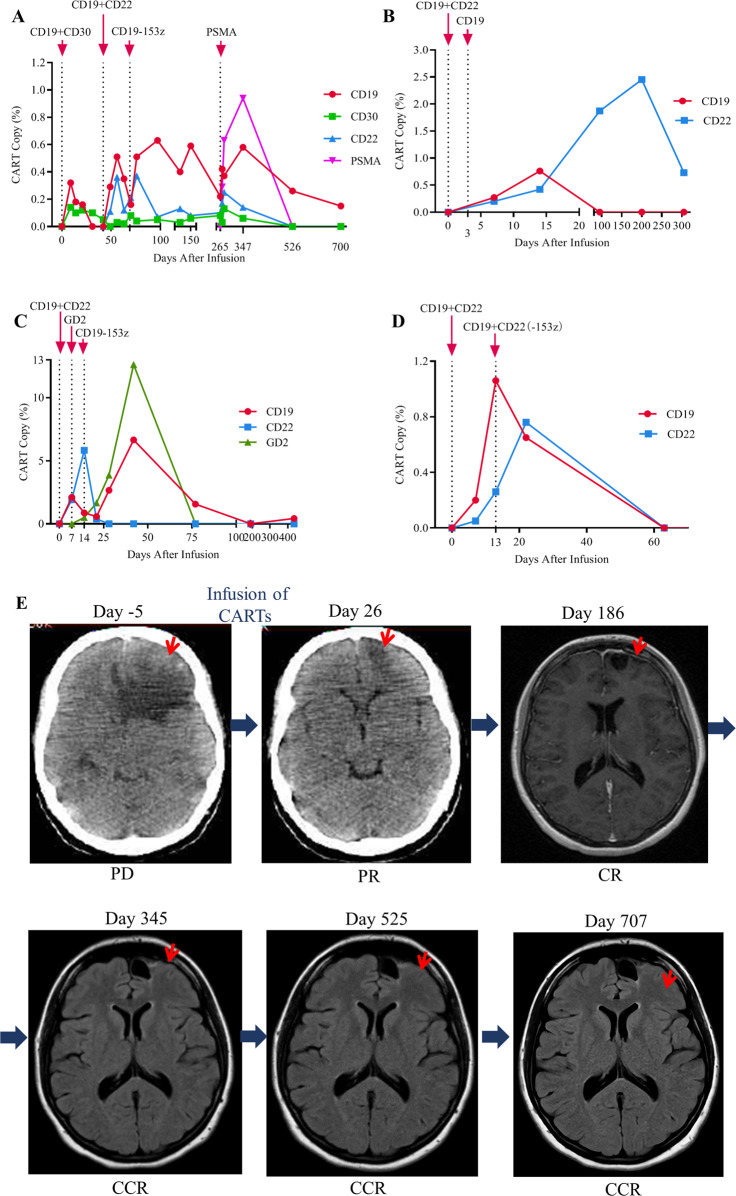
The kinetics of CAR-T cell expansion in the peripheral blood and scan images. **A** The copy number of CD19, CD22, CD30, and PSMA CAR-T cells in the peripheral blood of pt 1. **B** The copy number of CD19 and CD22 CAR-T cells in the peripheral blood of pt 2. **C** The copy number of CD19, CD22, and GD2 CAR-T cells in the peripheral blood of pt 3. **D** The copy number of CD19 and CD22 CAR-T cells in the peripheral blood of pt 4. **E** FDG-PET/CT and MRI scans of the brain lesions before and after CAR-T treatment of pt 1. Arrows point to the FDG-positive lesion in the left inferior frontal lobe before and after CAR-T. PD progressive disease, PR partial response, CR complete response, CCR continued complete response.

Pt 1 had a 32 × 26 × 31 mm lesion in the left inferior frontal lobe at the time of enrollment and experienced diarrhea, headache, and mild fever 37.5 °C (cytokine release syndrome (CRS) grade 1) at day 15 (d15) and d19 after CAR-T infusion. At d347, CD19, CD22, and PSMA CAR-Ts were still detected, illustrating persistence of the infused CAR-Ts. This was verified further as the CD19 CAR-T cells were still detectable on d526 (0.26%) and d701 (0.15%) (Fig. 1A). The fluorodeoxyglucose-positron emission tomography/computed tomography and magnetic resonance imaging scans showed that tumor decreased at d26 and disappeared completely by d186 following the first infusion (Fig. 1E). The patient has resumed normal daily activities without any CNS symptom and remains in continued complete remission (CCR) at the last follow-up (d725).

Pt 2 experienced fever of 38.7 °C on d5 without signs of infection (CRS grade 0–1). The CD19 (0.27%, 0.76%) and CD22 (0.2%, 0.42%) CAR-Ts were detected on d7 and d14, respectively. Importantly, the CD22 CAR-T cells were still detectable on d90 (1.87%), d200 (2.45%) and d305 (0.73%), but not the CD19 CAR-T cells (Fig. 1B). The pt remained in remission at d323 after CAR-T therapy.

Pt 3 received the primary (CD19 + CD22), booster (GD2), and consolidation (CD19-153z) CAR-T infusions at d0, d7, and d14, respectively, and had 0.43% CD19 CAR-T cells in the blood on d435 (Fig. 1C).

Pt 4 received a primary (CD19 + CD22) and a booster (CD19-153z + CD22-153z) infusions on d0 and d13, and CD19 and CD22 CAR-Ts were detected at 0.65% and 0.76% on d22 (Fig. 1D).

Pts 3 and 4 experienced no CRS after infusions. Pts 1, 3, and 4 had residual disease and obtained partial response after the primary infusion and achieved CR after the booster and consolidation CAR-T infusions. The only side effect was CRS of maximum grade 1, and no neurotoxicity was observed. All 4 pts have been in remission for >1 year and had no treatment-related toxicity. This study illustrates that combining different CAR-T cell products, based on individual tumor antigen profile, could improve tumor control without added toxicities.

Many studies have reported that, after CD19 CAR-T therapy, pts develop CD19-negative relapses. Besides, severe toxicities, such as CRS and neurotoxicity, have been haunting the currently approved CAR-T products. This 4SCAR2.0 study aims to use a combination of multiple CAR-T cells to avoid tumor antigen escape and obtain long-term remission in pts who do not have good conventional treatment options. The 4SCAR design is based on a low toxicity fourth-generation CAR technology and targets different antigens, including CD19, CD20, CD22, CD30, CD70, CD123, GD2, and PSMA. Because of our extensive experience with using fourth-generation CAR-T cells, approximately 800 pts with this type of product infused, which repeatedly showed very low frequency of CRS, mostly grade 0–1 and no greater than grade 2 in general, we were able to design a trial that allowed for combination of multiple CAR-T cells targeting different antigens based on a short production time (5–7 days) at reduced cost. Of note, this approach would not be feasible with currently approved products due to their different toxicity profile and high cost of manufacture; also the latter all target the same antigen thus using repeated infusions would be of limited benefit due to tumor antigen escape. Besides the safety profile, the 4SCAR2.0 study also demonstrated that multiple 4SCAR-T cells can co-exist in vivo for prolonged period of time, e.g., >200 days, in two of the four pts suggesting the establishment of CAR-T memory (Fig. 1A, B). Interestingly, the last infusion of the 4SCAR PSMA product in pt 1 on d265 resulted in an expansion of previously infused CD19, CD30, and CD22 CAR-T cells (Fig. 1A). Thus, there was no evidence of competitions among different CAR-Ts and rather it suggested a cooperative CAR-T interactions in vivo.

One critical factor to the success of CAR-T therapy for BCL pts is the condition of the pt’s immune system at the time of CAR-T engagement. Of note, prolonged cycles of chemotherapies and immune-suppressive regimens such as dexamethasone and lenalidomide often result in a highly suppressed immune state and have presented a major difficulty in generating good-quality CAR-T cells. Thus, for pt 2 enrolled in the study, we aimed to collect peripheral blood mononuclear cells prior to extended immunochemotherapy, at the time of relapse, and prior to reinduction chemotherapy. The good timing allowed us to obtain good-quality immune cells with a single apheresis sufficient for production of multiple CAR-T cell products, such as in pt 1.

Although long-term studies are necessary to evaluate this novel 4SCAR2.0 approach, any pt with a lethal disease is more concerned about imminent survival than long-term effects. Continued follow-up of these pts and additional enrollment are ongoing to fully assess efficacy and toxicity of this approach. Further studies are needed before using this treatment earlier in a disease course. Earlier use of 4SCAR-T cells targeting multiple antigens would be of particular interest for pts with high-risk disease due to genetics, or spread of disease, or frail pts who tolerate poorly extended chemotherapy.

In summary, a multiple CAR-T therapy regimen, including a primary, a booster, and an optional consolidation CAR-T infusions, resulted in achieving and maintaining prolonged remissions in four pts with rrBCL without short-term adverse effects. This combination 4SCAR strategy illustrated safety without any adverse effect. Long-term efficacy of this approach requires expanded investigation with more enrolled pts and continued follow-up. This innovative approach warrants additional investigation and further exploration of different CAR-T combinations.
